# Volatile Compound Markers in Beef Irradiated with Accelerated Electrons

**DOI:** 10.3390/molecules29050940

**Published:** 2024-02-21

**Authors:** Ulyana Bliznyuk, Polina Borshchegovskaya, Timofey Bolotnik, Victoria Ipatova, Aleksandr Kozlov, Alexander Nikitchenko, Irina Mezhetova, Alexander Chernyaev, Igor Rodin, Elena Kozlova

**Affiliations:** 1 Department of Physics, Lomonosov Moscow State University, Moscow 119991, Russia; 2 Skobeltsyn Institute of Nuclear Physics, Lomonosov Moscow State University, Moscow 119991, Russia; ipatova.vs15@physics.msu.ru; 3 Department of Chemistry, Lomonosov Moscow State University, Moscow 119991, Russiaigorrodin@yandex.ru (I.R.); 4 Department of Medical and Biological Physics, Sechenov First Moscow State Medical University, Moscow 119991, Russia; fillnoise@mail.ru (A.K.); waterlake@mail.ru (E.K.); 5 Department of Epidemiology and Evidence-Based Medicine, Sechenov First Moscow State Medical University, Moscow 119991, Russia

**Keywords:** electron beam irradiation, effective dose range, lipid oxidation, protein oxidation, reactive oxygen species, gas chromatography–mass spectrometry, volatile organic compounds, dose-dependent markers

## Abstract

This study focuses on the behavior of volatile organic compounds in beef after irradiation with 1 MeV accelerated electrons with doses ranging from 0.25 kGy to 5 kGy to find reliable dose-dependent markers that could be used for establishing an effective dose range for beef irradiation. GC/MS analysis revealed that immediately after irradiation, the chemical yield and accumulation rate of lipid oxidation-derived aldehydes was higher than that of protein oxidation-derived aldehydes. The nonlinear dose-dependent relationship of the concentration of volatile organic compounds was explained using a mathematical model based on the simultaneous occurrence of two competing processes: decomposition of volatile compounds due to direct and indirect action of accelerated electrons, and accumulation of volatile compounds due to decomposition of other compounds and biomacromolecules. A four-day monitoring of the beef samples stored at 4 °C showed that lipid oxidation-derived aldehydes, protein oxidation-derived aldehydes and alkanes as well as alcohol ethanol as an indicator of bacterial activity were dose-dependent markers of biochemical processes occurring in the irradiated beef samples during storage: oxidative processes during direct and indirect action of irradiation, oxidation due to the action of reactive oxygen species, which are always present in the product during storage, and microbial–enzymatic processes. According to the mathematical model of the change in the concentrations of lipid oxidation-derived aldehydes over time in the beef samples irradiated with different doses, it was found that doses ranging from 0.25 kGy to 1 kGy proved to be most effective for beef irradiation with accelerated electrons, since this dose range decreases the bacterial content without considerable irreversible changes in chemical composition of chilled beef during storage.

## 1. Introduction

With an increasing popularity of industrial food irradiation [1], the demand for irradiation markers calls for establishing reliable and easily detectable chemical compounds with a clear dependence of their concentration on the dose applied to a wide variety of meat and fish. Determining an effective irradiation dose range for meat and fish, which are composed of fats, proteins, and carbohydrates and are thus particularly susceptible to bacterial contamination, represents a challenge for researches, since the doses that can effectively inhibit bacteria may cause irreversible chemical changes that make foods unfit for consumption.

The effectiveness of food irradiation depends on many factors. While ionizing irradiation of frozen foods or foods containing preservatives or antioxidants is effective for a wide range of meat and fish as it significantly increases the shelf life of products [2,3], chilled meat and fish irradiation requires a special approach to selecting technological parameters, since intensive bacterial activity [4] and auto-oxidation [5] in these foods trigger significant physical and chemical changes during storage. Moreover, the intensity of biochemical processes occurring in chilled products during storage depends not only on the irradiation parameters, such as type of irradiation [6,7], irradiation dose [6,8], dose rate [7], and uniformity of absorbed dose distribution [9], but also on the initial concentration and spatial distribution of bacteria [10], types of microorganisms in the product [7], concentration of proteins and carbohydrates [11] actively consumed by microorganisms, storage temperature [12], pH values [13], and packaging type [14]. Thus, the effective dose range for chilled meat and fish, which is determined both by meat and fish properties as well as the technological regime of irradiation and storage, has to be established bearing in mind that the lower limit of the dose range would sufficiently suppress microorganisms and the upper limit would not lead to irreversible physical and chemical changes in the products [15].

The intensity of physical and chemical processes occurring in biological objects can be assessed by the presence and quantity of volatile organic compounds (VOCs), as they are particularly sensitive to any impact [16]. The type and quantity of VOCs reveal the composition of fats, proteins and carbohydrates in the product [17], as well as bacterial content and type of bacteria [18], since the intensity of autolysis, hydrolysis of lipids, fragmentation of amino acids, carbonylation of proteins, and microbial–enzymatic processes, which cause VOCs to appear, are determined by the type and concentration of bacteria in the product. The presence of oxygen in the product determines the rate of lipid and protein auto-oxidation as well as the decomposition of carbohydrates that manifest themselves through VOCs [19].

Studies using advanced GC/MS instrumentation have shown that significant changes in VOC content in meat and fish after irradiation [20] are associated with intensive oxidation of lipids and proteins by organic radicals [21], as well as products of lipid peroxidation [22], which are caused by both direct ionization and indirect action of irradiation through reactive oxygen species (ROS) [19,23]. Quantitative analysis of lipid oxidation products significantly contributes to the understanding of oxidative spoilage of various food products after exposure to various physicochemical factors [24]. Considering that the complexity of meat irradiation is exacerbated by the fact that beef is additionally acted upon by ferrous ions contained in myoglobin and hemoglobin that trigger a chain reaction in lipid oxidation [25], this study focuses on beef specifically to explore a wide variety of dynamic changes that may occur in irradiated chilled meat during storage [26].

The purpose of the study is to investigate the behavior of volatile organic compounds after irradiation and during storage of beef in order to find reliable dose-dependent markers that could be used on an industrial scale for establishing effective lower and upper dose limits for beef irradiation.

## 2. Results

**Figure 1:**
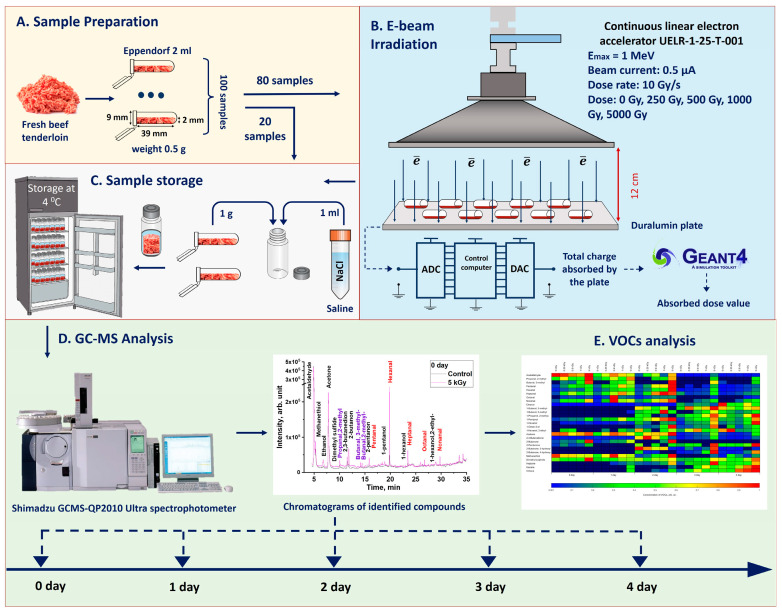
Stages of the research: (**A**) sample preparation; (**B**) electron beam irradiation; (**C**) sample storage in refrigerator; (**D**) chemical analysis on GC/MS; (**E**) identification and quantification of VOCs.

This study analyzes volatile compounds found in beef samples irradiated with 1 MeV electrons at doses of 250–5000 Gy using the GC/MS method. Beef samples stored at 4 °C in tightly sealed vials were tested for VOC profile each day for five days after irradiation. The volatile compounds in the beef samples were detected using chromatograms obtained during GC/MS processing, and the concentrations of VOCs were calculated taking into account the peak areas corresponding to different types of VOCs and the conversion coefficients obtained using the chromatographic analysis of standard volatile compound samples. The results of the analysis are presented in the form of a heat map to illustrate the effect of the dose and storage time on a great variety of VOCs. The stages of the experiment are shown in Figure 1.

### 2.1. Volatile Organic Compounds Identified in Irradiation Beef Meat

**Figure 2:**
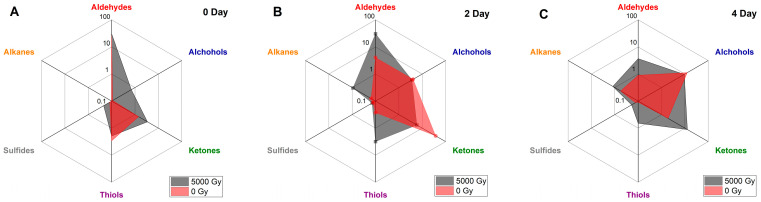
Volatile compounds identified in the non-irradiated beef samples and in the beef samples irradiated with 5 kGy measured on day 0 (**A**), day 2 (**B**) and day 4 (**C**) during storage time.

The GC/MS analysis revealed the presence of 19 to 27 compounds in the beef samples, including alcohols, aldehydes, ketones, alkanes, and sulfur-containing compounds, depending on the exposure dose and storage time. Figure 2 shows the detected classes of compounds, including the ratio between the summary concentrations of the compounds of different classes measured immediately after irradiation (Figure 2A) and on day 2 (Figure 2B) and day 4 (Figure 2C) of observation in the non-irradiated beef samples and in the samples irradiated with a dose of 5 kGy. The radar chart below illustrates the kinetics of the change in VOC composition over time: red shows the composition of VOCs identified in the non-irradiated beef samples, and gray shows the composition of VOCs identified in the beef samples irradiated with 5 kGy.

The most common compounds identified in the beef samples during the observation are aldehydes and alcohols, which are also commonly found in other types of meat and poultry, such as pork [27], lamb [28], rabbit [29], chicken [30], turkey [31], and duck [32]. Immediately after irradiation, alcohols and sulfur-containing compounds that were not detected in the non-irradiated beef samples were recorded in the beef samples irradiated with different doses, and there is a certain dependence of concentration on the irradiation dose. At the beginning of the study, some compounds, later found in both non-irradiated and irradiated beef samples, were absent and appeared gradually over time (Figure 2A). At the same time, some ketones were identified in the non-irradiated and irradiated beef samples within two days after irradiation (Figure 2B) to disappear over the storage time. Interestingly, in the irradiated beef samples, all the aldehydes that were detected immediately after irradiation could also be found during all four days of observation (Figure 2). Most of the alcohols, on the other hand, were detected after two days of storage in both non-irradiated and irradiated samples (Figure 2B). However, none of the alkanes was detected immediately after irradiation in non-irradiated or irradiated beef samples (Figure 2A). Two sulfur-containing compounds that were detected on day 0 in both non-irradiated and irradiated beef samples could also be found during all four days of storage (Figure 2). Half of the ketones that were found in the beef samples during the experiment were detected on day 0, while the remaining ketones appeared over time (Figure 2).

### 2.2. Analysis of Volatile Organic Compounds in Beef Immediately after Irradiation

**Figure 3:**
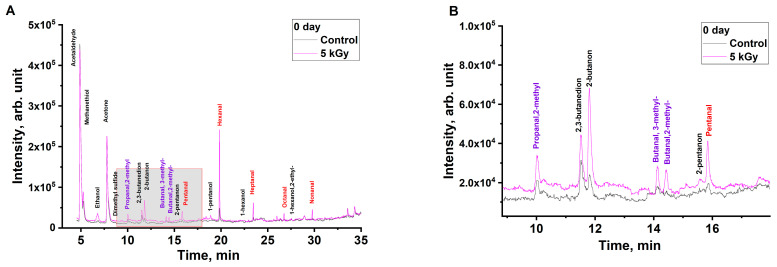
Chromatograms of non-irradiated beef sample shown as a black line and the sample irradiated with 5 kGy shown as a violet line. Lipid oxidation products are highlighted in red and protein oxidation products are highlighted in purple. (**A**)—General overview, (**B**)—zoom-in on shaded area.

Non-irradiated and irradiated beef samples were subjected to GC/MS analysis one hour after irradiation with accelerated electrons. Figure 3 shows the chromatograms of an non-irradiated beef sample and a sample irradiated with 5 kGy.

The beef samples irradiated with 5 kGy demonstrate a greater number of peaks and an increase in the peak areas compared to the non-irradiated samples, which can be explained by the oxidation of lipids and proteins in the beef samples during irradiation. As can be seen from Table 1 below, an increase in irradiation dose causes a greater number of VOCs to appear.

Most aldehydes (pentanal, hexanal, heptanal, octanal, nonanal), ketones (2,3-butandione, 2-pentanone), and alcohols (ethanol, 1-pentanol, 1-hexanol) show a dramatic increase in the concentration in the samples irradiated with 0.25 kGy and 0.5 kGy followed by a smoother rise in the concentration, while the irradiation with 5 kGy yields a concentration of VOCs that exceeds the control values by a factor of 1.9–7. In the case of 2-butanone ketone, an almost linear increase in concentration is observed with an increase in the irradiation dose. Importantly, while aldehydes, which are oxidation derivatives of lipids, were present in the non-irradiated beef samples (Figure 3, highlighted in red), aldehydes butanal,2-methyl- and butanal,3-methyl- (Figure 3, highlighted in purple), which are oxidation derivatives of proteins, were detected in the irradiated samples but not in non-irradiated ones.

It was found that the dose–effect relationship of VOC concentration was nonlinear, which, as our mathematical model shows, can result from two competing processes: the decomposition and accumulation of VOCs after irradiation as a result of the decomposition of other VOCs and biomacromolecules, such as fatty acids, proteins and carbohydrates. According to the model developed and published by our team [33], a volatile compound *A* present in the food sample at concentration *C^A^* is caused by the ionizing radiation to decompose at a rate *k_A_*, which is the number of compounds *A* decomposed per unit of absorbed dose (Gy^−1^). Under irradiation, compound *A* can transform into other volatile organic compounds of different fractions, depending on the type of the resulting compound.

Suppose that during irradiation, only a fraction *q* of the concentration of compound *A* decomposes into volatile compound *B*, with the value of *q* in the dose range independent of the irradiation dose and determined only by the type of compound *B*, which in turn also decomposes under the action of irradiation at rate *k_B_* (Gy^−1^). Thus, the *C^B^* concentration of compound *B* on the one hand increases as it is transformed from compound *A*, and on the other hand decreases as a result of decomposition caused by irradiation. The change in the concentrations of compounds *A* and *B* with an increase in the irradiation dose can be expressed as follows:

(1) $$\frac{dC^{A}}{dD}=-k_{A}C^{A}$$

(2) $$\frac{dC^{B}}{dD}=\frac{-dC^{A}}{dD}-k_{B}C^{B}D$$

Suppose that before irradiation, the concentration of compounds $C^{A}\left(D=0\right)=C_{0}^{A}$ and $C^{B}\left(D=0\right)=C_{0}^{B}$. The solution of Equations (1) and (2) can be expressed as follows:

(3) $$C^{A}\left(D\right)=C_{0}^{A}e^{-k_{A}D}$$

(4) $$C^{B}\left(D\right)=C_{0}^{B}e^{-k_{B}D}-\frac{qk_{A}C_{0}^{A}}{k_{B}-k_{A}}\left(e^{-k_{A}D}-e^{-k_{B}D}\right)$$

Since compound *A* can not only decompose into different compounds but can also occur in the food sample during irradiation when another volatile compound or biomacromolecules decompose as a result of irradiation, the dose dependence of *C^B^* concentration can be expressed as follows:

(5) $$C^{B}\left(D\right)=C_{0}^{B}e^{-k_{B}D}-\frac{qk_{A}C_{0}^{A}}{k_{B}-k_{A}}\left(e^{-k_{A}D}-e^{-k_{B}D}\right)+f\left(D\right)$$

where the function *f*(*D*) depends linearly on the dose ranging from 0.25 kGy to 10 kGy. Suppose that *f*(*D*) depends linearly on the dose *D* and *f*(*D*) *= gD*, where *g* (Gy^−1^) is the proportionality factor.

**Figure 4:**
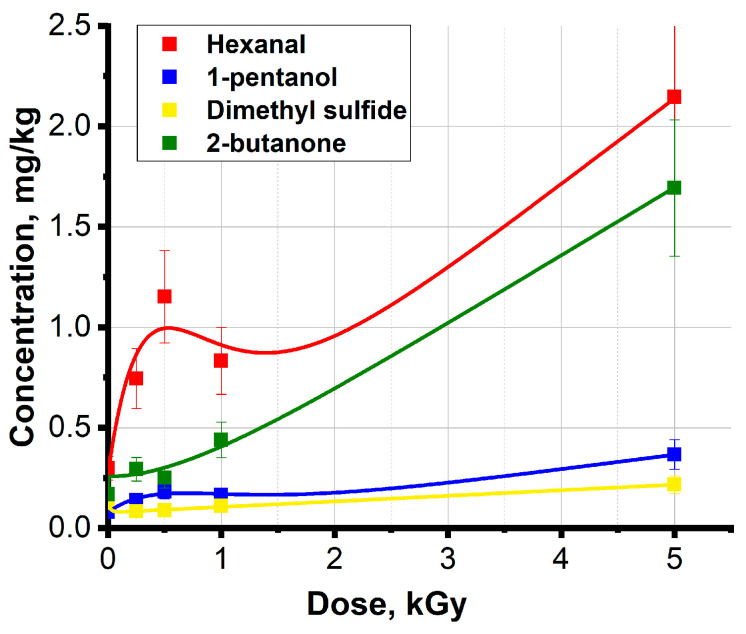
The dependencies of the concentrations of alcohol, aldehyde, ketone and sulfur-containing compound in the beef samples on the irradiation dose and the corresponding functions, calculated using Formula (5).

Figure 4 shows the functions calculated using Formula (5), which approximate the dependencies of concentrations of different classes of VOCs, such as hexanal, 1-pentanol, 2-butanone and dimethyl sulfide, which are commonly identified in irradiated meat and fish. Table 2 presents the approximation parameters obtained using Formula (5). High values of correlation coefficients and low standard error SE prove the validity of the mathematical model proposed by our team.

Figure 4 shows the typical behavior of VOC concentrations from the irradiation dose: a significant increase in the compound concentration in the beef samples irradiated with 5 kGy, which can be associated with a large number of chemical bond breaks in long organic chains leading to the formation of VOCs. All the dependencies presented in Figure 4 have a local minimum, which is a sign of competition between decomposition and accumulation of VOCs. Hexanal and 1-pentanol have a local minimum in the region of 1 kGy, whereas a local minimum for dimethyl sulfide and 2-butanone shifts to around 250 Gy and 500 Gy, accordingly. Since the model was developed specifically for the pair of compounds in which one compound is an oxidation product of the other one, it does not take into account a wider range of channels through which each compound may, with a different probability, be formed and decomposed.

### 2.3. Analysis of Volatile Organic Compounds in Irradiated Beef Meat by GC/MS during Storage Time

During storage for four days, GC/MS analysis identified eight compounds more than on the day of irradiation. A total of 27 different volatile organic compounds were recorded: eight aldehydes (acetaldehyde (ethanal), propanal,2-methyl-, butanal,3-methyl-, pentanal, hexanal, heptanal, octanal, nonanal); six ketones (acetone, 2,3-butandione, 2-butanone, 2-pentanone, 2-butanone,3-hydroxy-, 2-butanone,3-hydroxy-); eight alcohols (ethanol, 1-butanol,3-methyl-, 1-butanol,2-methyl-, 1-propanol,2-methyl-, 1-pentanol, 1-hexanol, 1-octen-3-ol, 1-hexanol,2-ethyl-); two sulfur-containing volatiles (methanethiol, dimethyl sulfide); three alkanes (heptane, hexane, octane) and thiols. In similar works by other authors [20] which analyzed VOCs in beef after irradiation, other classes of compounds such as pyrazines, alkenes, furans, and phenols were detected, which may be due to the different sensitivity of analytical instruments used for VOC identification and quantitative analysis, and beef properties, such as different intramuscular fat content [34], or different types of fodder [35].

**Figure 5:**
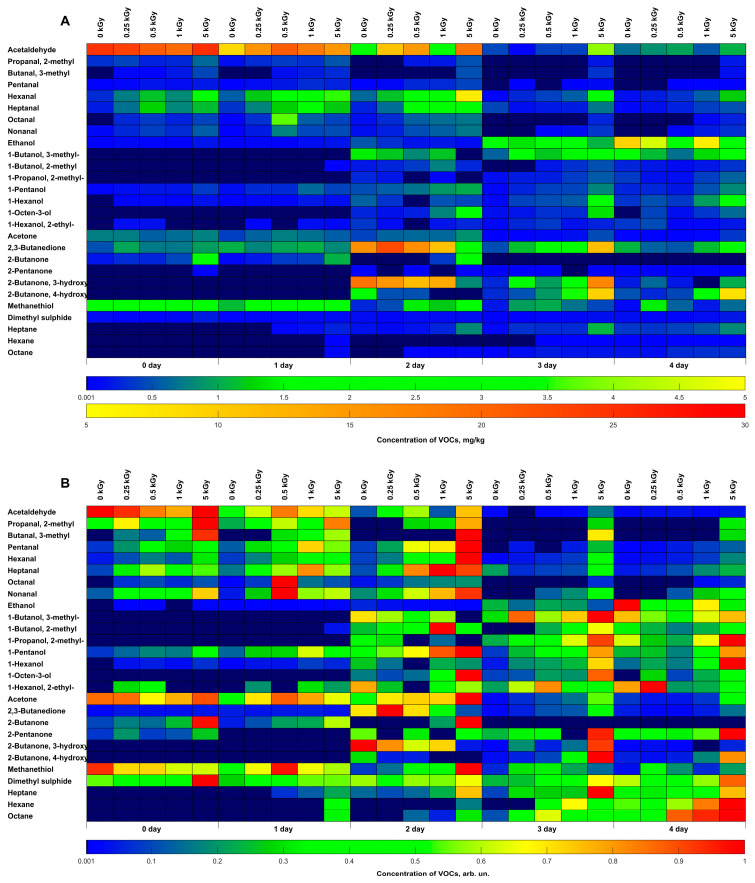
Heat map of volatile organic compound concentration in beef irradiated with different doses during four days of storage: (**A**) in mg/kg, (**B**) in relative units. Dark-blue cells show that the compound was not detected.

Figure 5 shows the concentrations of VOCs in beef irradiated with different doses and non-irradiated beef samples that were identified during four days of storage. Various VOCs are plotted on the ordinate axis of the heat map, and the data on the abscissa axis are grouped by irradiation dose and storage time. The concentrations of VOCs (mg/kg) and the concentrations normalized to the maximum value of each compound are shown in Figure 5A,B, respectively. Quantitative VOC monitoring data in non-irradiated and irradiated beef samples can be found in Supplementary Table S1.

A number of compounds, such as 1-butanol,3-methyl-, 1-butanol,2-methyl-, propanol,2-methyl-, 1-octen-3-ol, 2-butanone,3-hydroxy-, 2-butanone,4-hydroxy-, heptane, hexane, and octane, were absent during the first 2 days of storage in both non-irradiated and irradiated beef samples. However, on day 2 after irradiation, all the abovementioned compounds were detected in the stored samples. It can also be noted that the maximum VOC concentrations were observed for most compounds in the samples irradiated with 5 kGy, which indicates a significant effect of the applied dose on the beef properties due to oxidation caused by ROS occurring in the samples during and after irradiation.

The heat maps show that different classes of compounds prevail depending on the day of storage. While a high content of aldehydes and methanethiol was observed from day 0 to day 2 after irradiation, and ketones as well as alcohols increased in the concentration from day 2 to day 3 and from day 2 to day 4, respectively, alkanes showed a dose-dependent growth from day 3 to day 4 after irradiation. Since VOCs determine beef odor, it can be concluded that the prevalence of aldehydes and sulfur-containing compounds causes beef to exude a specific odor immediately after irradiation. A specific smell of beef after 3–4 days of storage upon irradiation is the result of an increased concentration of alcohols and alkenes.

**Figure 6:**
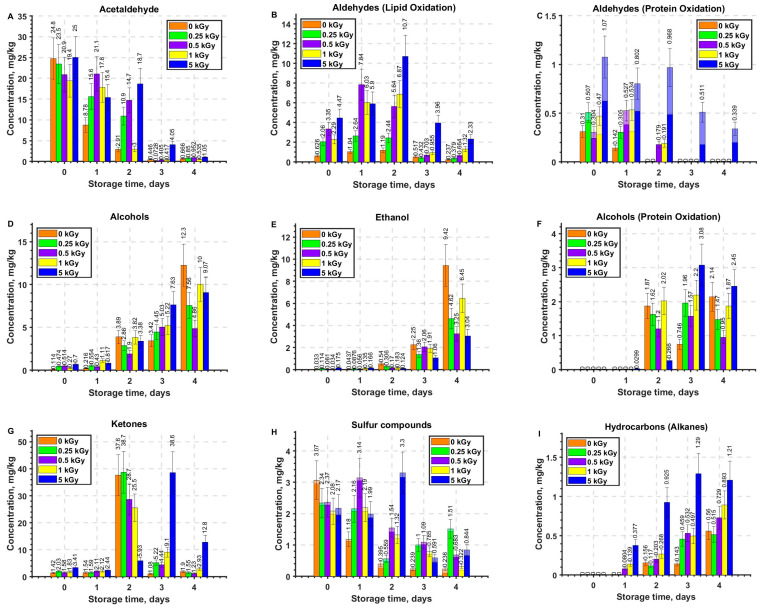
Dependencies of concentration (mg/kg) of acetaldehyde (**A**), summary concentration of aldehydes that are lipid oxidation derivatives (**B**), summary concentration of aldehydes that are protein oxidation derivatives (lighter colors represent the summary concentration of butanal,3-methyl- and butanal,2-methyl-, darker colors represent the concentration of propanal,2-methyl-) (**C**), alcohols (**D**), ethyl alcohol (**E**), alcohols that are products of protein oxidation (**F**), ketones (**G**), sulfur-containing compounds (lighter colors represent the concentration of dimethyl sulfide and darker colors represent the concentration of methanethiol (**H**), and alkanes (**I**) in the beef samples on storage time.

Figure 6A–I show the kinetics of the concentrations of different classes of volatile compounds, such as aldehydes, excluding acetaldehyde, as well as alcohols, ketones, alkanes, acetaldehyde, ethanol, methanethiol, dimethyl sulfide and butanal,3-methyl- in the beef samples irradiated with different doses as well as in non-irradiated samples. The time dependencies of the concentrations of acetaldehyde, butanal,3-methyl-, propanal,2-methyl, acetone, ethyl alcohol, and sulfur-containing compounds methanethiol and dimethyl sulfide in the beef samples are plotted individually, since these compounds show dramatically different behavior depending on the dose and storage time, which will be discussed further in this article.

Experimental results show that compounds within each individual class have the same dependencies of the concentrations on the time and dose, so for convenience, further discussion will take into account the mechanisms specific for each class.

#### 2.3.1. Aldehydes

Despite the fact that the concentration of acetaldehyde in irradiated and non-irradiated samples decreased during storage (Figure 6A), this compound was the predominant aldehyde in beef for all irradiation doses at different storage time as a result of decomposition of various fatty acids and alcohols present in beef [36]. The nonlinear decrease in acetaldehyde concentration in irradiated and non-irradiated beef samples with time can be explained by its decomposition due to high reactivity and high oxidation rate in the presence of oxygen. The irradiation dose also had a nonlinear effect on the behavior of acetaldehyde throughout beef monitoring. It should also be noted that the acetaldehyde concentration plummeted on day 3 after irradiation after gradually subsiding within the first two days of storage.

##### Lipid Oxidation Aldehydes

According to previous studies [20], degradation products of unsaturated fatty acids and straight-chain aldehydes, such as pentanal, hexanal, heptanal, octanal, and nonanal, are responsible for the specific odor of meat products after irradiation. Oxidation products of lipids showed a similar dependence of the concentration on the dose and storage time: an increase in concentration on day 1 and day 2 after irradiation followed by a sharp decline for the remaining period of monitoring (Figure 6B). Interestingly, the time dependencies of both individual aldehydes and summary aldehyde concentration were clearly influenced by the irradiation dose: the higher the irradiation dose, the higher the maximum value of aldehyde concentrations recorded in the beef samples. Moreover, the peak chemical yield of aldehydes formed by lipid oxidation shifted towards longer observation time with an increase in the irradiation dose. It should be noted that the maximum and most prolonged effect of the release of aldehydes formed during lipid oxidation was observed in the beef samples irradiated with a dose of 5 kGy.

##### Protein Oxidation Aldehydes

An oxidation product of isoleucine amino acid branched aldehyde butanal,3-methyl [35], a constituent of protein molecules, was not detected in the non-irradiated beef samples throughout the entire period of the experiment, which corresponds to similar experiments involving beef [37]. In the samples irradiated with 0.25 kGy, butanal,3-methyl was only detected immediately after irradiation, and the concentration of this aldehyde was 0.08 mg/kg. In the samples irradiated with 0.5 kGy and 1 kGy, the concentration of butanal,3-methyl ranged from 0.06 mg/kg to 0.2 mg/kg, with the peak concentration observed on day 1 after irradiation, while no traces of butanal,3-methyl were detected further. The concentration of butanal,3-methyl in the samples irradiated with 5 kGy had a maximum on day 2 of monitoring, and after four days of storage, this aldehyde was still present in the sample, although its concentration decreased during the remaining two days of monitoring (Figure 6C). Aldehyde butanal,2-methyl was only detected immediately after irradiation and it was not found during a further four days of monitoring.

The concentration of aldehyde propanal,2-methyl, formed both by oxidation of the alcohol propanol, amino acid isoleucine and amino acid valine [38], decreased nonlinearly with the storage time. While propanal,2-methyl was initially present both in non-irradiated and all irradiated beef samples, its concentration subsided at a rate determined by the applied irradiation dose: the higher the dose, the more time was required for propanal,2-methyl to disappear. The concentration of propanal,2-methyl in the samples irradiated with 5 kGy had a maximum immediately after irradiation, and after four days of storage, this aldehyde was still present in the beef samples irradiated with 5 kGy, although its concentration decreased during the remaining four days of monitoring (Figure 6C). It should be noted that while protein oxidation aldehydes registered in the beef samples irradiated with doses ranging from 0.25 kGy to 1 kGy were detected only within 2 days after irradiation, the beef samples irradiated with 5 kGy contained aldehydes during four days of observation (Figure 6C).

The aldehydes, which are lipid oxidation derivatives detected in the irradiated beef samples, showed dramatically different dependence on the storage time compared to aldehydes, which are protein oxidation products: in contrast to a well-defined concentration peak of aldehydes formed from lipids during first two days after irradiation depending on the dose applied, after a slight growth in the concentration of the aldehydes formed from proteins during and immediately after irradiation, a gradual reduction in the value was observed throughout further four days of monitoring.

#### 2.3.2. Alcohols

The total concentration of alcohols in all non-irradiated and irradiated beef samples increased with the storage time, and while it was marginally low immediately after irradiation and on day 1 of storage, the values rocketed on day 2 and continued growing during a further two days of monitoring (Figure 6D). Straight-chain alcohols 1-pentanol and 1-hexanol are formed by oxidation of unsaturated fatty acids, similar to straight-chain aldehydes, while branched-chain alcohols 1-butanol,3-methyl-, 1-butanol,2-methyl-, 1-propanol,2-methyl-, 1-octen-3-ol and 1-hexanol,2-ethyl- can be accumulated as a result of the decomposition of proteins due to bacterial activity [39] or other biochemical processes [38].

The main contribution to the concentration of alcohols was made by ethanol (Figure 6E), which is a product of glycogen hydrolysis initiated by microbial enzymatic transformations [40]. The concentration of this compound in the non-irradiated beef samples increased exponentially from 0.033 mg/kg to 9.42 mg/kg during four days of storage, which is an indication of an increase in bacterial activity in the non-irradiated beef samples. A steady increase in ethanol concentration was observed in the beef samples irradiated with doses ranging from 0.25 kGy to 5 kGy, and the rate at which ethanol increased slowed as the irradiation dose went up, which is a sign of the inactivation of microorganisms by irradiation. Therefore, the higher the irradiation dose, the lower the concentration of microorganisms in beef and the lower the concentration of ethanol.

The concentration of straight-chain alcohols pentanol-1 and hexanol-1 showed a nonlinear dependence on storage time in the non-irradiated and irradiated beef samples. In the non-irradiated samples, the maximum concentrations of pentanol-1 and hexanol-1 (0.5–0.6 mg/kg) were observed on day 2 of storage. In all irradiated samples, pentanol-1 peaked on day 2–3 of storage for all irradiation doses. The concentration of hexanol-1 in irradiated beef samples increased nonlinearly with storage time (from 0.07–0.16 to 0.49–2.24 mg/kg) for all the doses. The higher the irradiation dose, the higher the concentration of this compound was detected in the beef samples, which is a sign of predominant accumulation of pentanol-1 and hexanol-1 due to oxidation caused by reactive oxygen species, whose concentration typically goes up with an increase in irradiation dose.

Despite its low concentration, 1-octen-3-ol (0.1–1.5 mg/kg), with a specific mushroom flavor odor, is a key flavor component of beef meat [20]. Alcohol 1-octen-3-ol may be a degradation product of linoleic fatty acid or a reduction product of carbonyl compounds [41]. In this study, 1-octen-3-ol was detected in all samples from two days of monitoring onwards: its concentration in the non-irradiated samples and the samples irradiated with 0.25 kGy and 0.5 kGy stood at the level of 0.1–0.4 mg/kg and did not change until the end of observation. In contrast, for doses of 1 kGy and 5 kGy an increase in the concentrations on day 2 of storage was three and six times higher compared to the control values, respectively, and further there was a decrease in the concentration of 1-octen-3-ol to 0.4 mg/kg and 0.7 mg/kg, for 1 kGy and 5 kGy, respectively, as this alcohol started to decompose.

In the remaining branched-chain alcohols 2-methyl-1-butanol, 3-methyl-1-butanol and 2-methyl-1-propanol, which are protein oxidation products, a nonlinear increase in the concentrations with the storage time was observed for all irradiation doses. It should be noted that branched-chain alcohols were absent in all beef samples during two days of monitoring (Figure 6F). Therefore, a sharp growth in the concentration of these alcohols observed on day 2 to day 4 of storage after irradiation may be a sign of bacterial activity as well as oxidation occurring in the beef samples initiated by ROS present in both non-irradiated samples and to a greater extent in the irradiated samples throughout the observation period. At the same time, the concentration of alcohols formed by protein degradation detected in the beef samples irradiated with 5 kGy on day 2 of minoring was 10 times higher compared to ones in the beef samples irradiated with other doses (Figure 6F). Thus, the concentration of alcohols from protein degradation is also an indicator of the effective microbial suppression in meat samples by irradiation.

#### 2.3.3. Ketones

The total concentration of all ketones detected in the beef samples showed a dramatic increase on day 2 after irradiation for the doses ranging from 0 to 1 kGy reaching the peak of 25.5 mg/kg to 37.6 mg/kg depending on the dose, while the maximum concentration of ketones detected in the beef samples irradiated with 5 kGy was achieved on day 3 after irradiation and amounted to 35.6 mg/kg (Figure 6G). The main contribution to the total concentration of ketones after irradiation was made by 2-butanone,3-hydroxy- and 2,3-butandione, which are responsible for a specific oily odor of meat [20]. According to the literature [42], 3-hydroxy-2-butanone and 2,3-butanedione are accumulated in meat products as a result of oxidation of the alcohol 2,3-butanediol, which is a product of fatty acid oxidation, through the decomposition of dicarbonyl and hydroxycarbonyl and through the autolysis of the polysaccharide glycogen, which is present in muscle tissue [43]. Since no alcohol 2,3-butanediol was identified in any of the beef samples during the entire period of storage, 2-butanone,3-hydroxy- and 2,3-butandione were formed as a result of glycogen autolysis in the presence of hydrolytic enzymes catalyzing the decomposition of glycogen.

#### 2.3.4. Sulfur-Containing Compounds

The sulfur-containing compounds methanethiol and dimethyl sulfide identified in the beef samples are derived from the degradation of sulfur-containing amino acids such as methionine, cysteine and cystine [44], which are the building blocks of proteins. Dimethyl sulfide, detected in the non-irradiated and irradiated beef samples, was present at low concentrations (0.2 mg/kg or less) throughout the entire period of storage (Figure 6H). Though the concentration of dimethyl sulfide is relatively low, it is responsible for a rather unpleasant odor that is frequently identified in irradiated meat products [37]. It should be noted that the concentrations of this compound in beef samples irradiated with doses ranging from 0.25 kGy to 1 kGy were close to the control values throughout the observation time. The samples irradiated with 5 kGy showed a statistically significant 1.1–2.8-fold increase in dimethyl sulfide concentration compared to the controls throughout the entire period of monitoring.

The concentration of methanethiol, which has a putrid odor [20], in the non-irradiated beef samples decreased exponentially from 3.0 mg/kg to 0.1 mg/kg during the whole observation time (Figure 6H). In all irradiated beef samples, this compound showed a non-monotonic dependence of concentration on the storage time. Thus, in the samples irradiated with 0.25 kGy, the concentration of methanethiol decreased from 2.3 mg/kg to 0.5 mg/kg during the first two days to increase to 1.4 mg/kg on day 4 of monitoring. In the samples irradiated with 0.5 kGy and 1 kGy, the concentration of methanethiol was at the level of 2–3 mg/kg for the first two days and then demonstrated a nonlinear decrease with the storage time to 0.6 and 0.2 mg/kg, respectively.

In the samples irradiated with 5 kGy, for the first two days there was no significant change in the concentration of methanethiol (2.0–1.9 mg/kg), but on day 3 the concentration increased almost 1.5 times to decrease to 0.5–0.7 mg/kg on day 4 after irradiation. The monotonic decrease in methanethiol concentration throughout the storage time in the non-irradiated samples testifies to the gradual decomposition of methanethiol as a result of meat oxidation. Non-monotonic kinetics of methanethiol concentration in the irradiated samples is a sign of competition between accumulation of this compound triggered by protein oxidation, and decomposition of methanethiol as it is oxidized by reactive oxygen species.

#### 2.3.5. Alkanes

The concentration of the identified alkanes heptane, hexane and octane, which are a combination of two organic radicals formed during the oxidation of fatty acids, increased nonlinearly in the irradiated beef samples with an increase in the storage time, and none of these compounds was detected in the non-irradiated beef samples (Figure 6I). Alkanes were detected in the beef samples irradiated with doses ranging from 0.5 kGy to 5 kGy one day after irradiation, and the higher the dose, the higher concentration of these compounds. In the beef samples irradiated with 0.25 kGy, alkanes were detected two days after irradiation, and their concentration was at the level of the non-irradiated samples. Between day 2 and day 3 after irradiation, the concentration of alkanes in the samples irradiated with 0.25 kGy increased, and on day 3 after irradiation it was close to that of the samples irradiated with 0.5 kGy and 1 kGy. On day 4 after irradiation, the concentration of alkanes in the non-irradiated samples increased to the level of the samples irradiated with 0.25 kGy, so alkane concentration in the non-irradiated samples did not statistically differ from the concentration in the samples irradiated with 0.25 kGy. The highest concentration of alkanes (1.29 mg/kg) in the samples irradiated with 5 kGy registered on observation day 3 was dramatically different from the concentration of alkanes in all other irradiated samples, which ranged from 0.46 mg/kg to 0.53 mg/kg.

### 2.4. Oxidation Markers in Irradiated Beef during Storage

**Figure 7:**
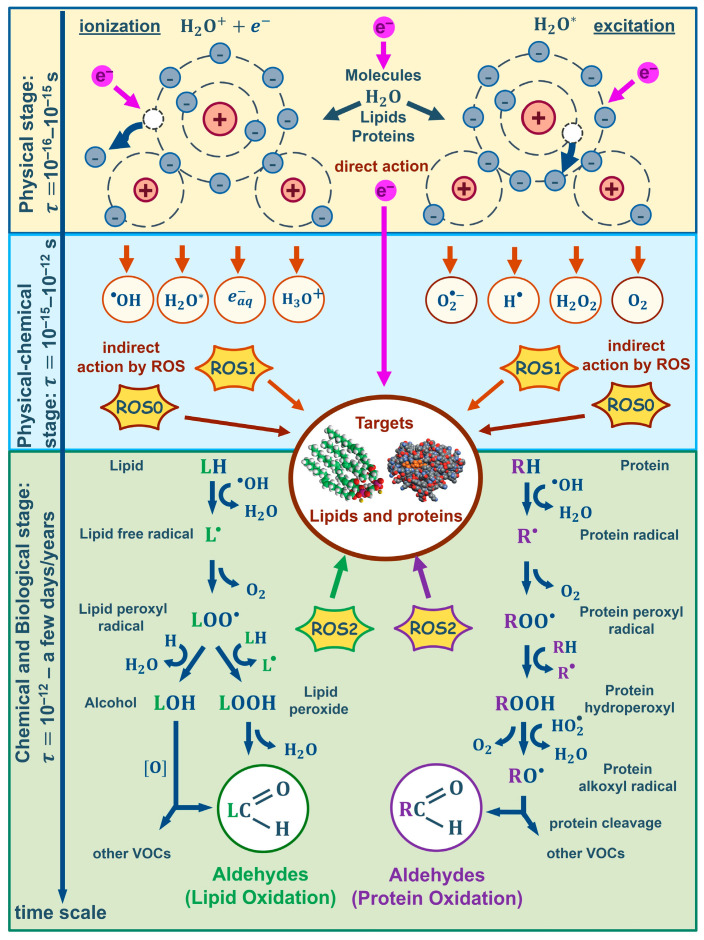
Stages of lipid and protein oxidation in beef due to irradiation with accelerated electrons.

The impact of electron beams on biological tissue of meat as a result of both direct ionization of atoms and molecules by accelerated electrons as well as indirect action of electrons lead to biological dose–effect impacts in biomacromolecules. When exposed to accelerated electrons, chemical bonds in lipid and protein molecules break to initiate changes in molecule structure or charge distribution—polarity of molecules. The physical stage of radiation lasting for $\tau_{phys}=10^{-16}÷10^{-15}$ s [45] leads to the creation of ions and excited molecules, while ROS_1_ species $O_{2},\;O_{2}^{1},\;O_{2}^{*-},\;{OH}^{*},\;{OH}^{+},\;{OH}^{-},\;H_{2}O_{2},\;H_{2}O_{2}^{-}$, occurring as a result of water radiolysis, during physical and chemical stage of the irradiation $\tau_{phys/chem}=10^{-15}÷10^{-12}$ s actively interact with lipids, bringing about oxidative stress that initiates lipid peroxidation, triggering chain destructive reactions in the cell lipids and forming lipid radicals L*, LO*, as well as lipid hydroperoxides LOOH—ROS_2_ [24]. ROS_1_ species also interact with amino acids, peptides and proteins, leading to both reversible and irreversible oxidation of proteins with the formation of protein peroxyl radicals (ROS_2_), causing disruption, modification, carbonylation, oxidation and fragmentation of the primary, secondary and tertiary structure of protein molecules. The biochemical stage of irradiation lasts from $\tau_{chem/biol}=10^{-12}$ s to several days and even years [45]. Figure 7 shows the stages of accelerated electron exposure for target lipids and proteins in meat samples.

Biochemical changes in the beef samples initiated by lipid and protein radicals keep occurring from a few seconds to a few days after irradiation. According to the experimental results obtained in our study, the dose–effect impact on the formation of lipid oxidation markers pentanal, hexanal, heptanal, octanal, and nonanal was detected throughout the four-day monitoring (Figure 6B): the total concentration of aldehydes formed from lipid oxidation was higher with higher doses absorbed by the beef samples on each day of observation. Moreover, with an increase in the irradiation dose, the concentration peak of aldehydes occurred on later days of storage. It can be concluded that the direct action of accelerated electrons and indirect action of primary ROS_1_ only affects the aldehyde concentration immediately after irradiation, while the secondary and tertiary ROS_2_, whose concentration is higher the higher the irradiation dose, is evident throughout the entire period of storage. During storage in packaging that is not very tight, both non-irradiated and irradiated chilled meat is inevitably affected by background ROS_0_ species, which are formed in the presence of oxygen. This explains why marginal concentrations of aldehydes formed from lipids can be traced in the non-irradiated beef samples.

**Figure 8:**
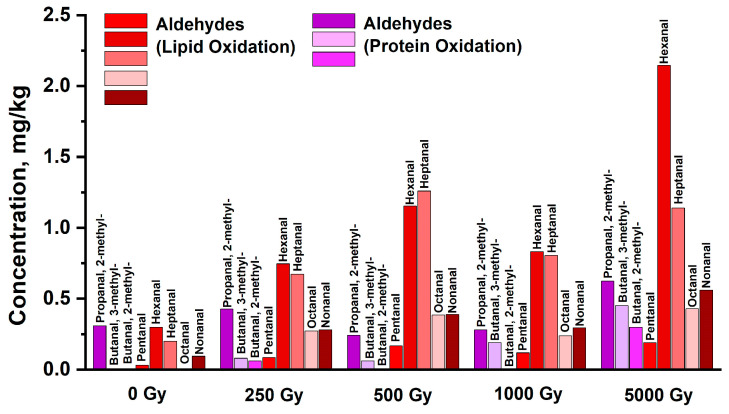
Histogram of concentrations of aldehydes detected immediately after irradiation in the non-irradiated and irradiated beef samples: protein oxidation aldehydes are shown in different tints of violet and lipid oxidation aldehydes are highlighted by the red palette.

Immediately after exposure to accelerated electrons, the protein oxidation aldehydes butanal,2-methyl, butanal,3-methyl and propanal,2-methyl were detected in the beef samples, and in contrast to the lipid oxidation markers that are present in the non-irradiated samples, butanal,2-methyl and butanal,3-methyl were detected only in the irradiated beef samples, with their concentration becoming commensurate with that of lipid-derived aldehydes only when irradiated with 5 kGy (Figure 8). Aldehyde butanal,2-methyl was detected only immediately after irradiation and never traced in the further four days of monitoring. In contrast, the dose–effect impact on the formation of protein oxidation marker butanal,3-methyl was observed throughout the whole period of storage (Figure 6C). Immediately after irradiation and during the first two days of monitoring, butanal,3-methyl concentration was higher with higher irradiation dose. While immediately after irradiation, butanal,3-methyl was detected in all the samples irradiated with different doses, after the first day of storage, it registered only in the samples irradiated with 0.5 kGy, 1 kGy and 5 kGy, and during the remaining days of monitoring, butanal,3-methyl was detected only in the beef samples irradiated with 5 kGy. Bearing in mind that butanal,3-methyl showed a clear dose dependence over time, this aldehyde can serve as a potential marker of protein oxidation in beef. Judging by the dose dependence of lipid and protein oxidation markers measured immediately after irradiation, it can be concluded that lipid oxidation is observed at lower doses than protein oxidation in beef. A similar effect of ROS on blood in vivo is observed when oxidation of blood cell lipids occurs at a lower concentration of ROS than carbonylation of protein molecules in blood cell membranes [46].

During the monitoring, a clear dose dependence of the concentration of hydrocarbons formed by combining two lipid radicals was detected (Figure 6I): on days 1–4 of the monitoring, and their concentration was higher the higher the dose absorbed by the beef samples. At the same time, immediately after irradiation, alkanes were not detected in either non-irradiated or in irradiated beef samples. Since the higher the dose, the more ROS_1_ occur in the product and the more organic ROS_2_ radicals are formed, it can be concluded that alkanes are markers of multistage oxidative processes of lipids in beef after irradiation.

Unlike irradiation markers of lipid and protein oxidation, ethanol was detected in all beef samples on all five days of monitoring. Beef monitoring after irradiation revealed a clear dependence of ethanol concentration on the irradiation dose: the higher the dose, the lower the rate at which ethanol concentration grew in the beef samples during storage and the lower the ethanol concentration recorded (Figure 6E). Ethanol—a product of glycogen hydrolysis initiated by microbial enzymatic transformations—is a marker of bacterial content in meat during storage: the higher the ethanol concentration, the more bacteria are present in meat. Since ethanol was found in the beef samples irradiated with 5 kGy, though on day 4 its concentration was three times lower than non-irradiated samples, it is clear that even the highest irradiation dose did not fully inhibit bacteria in the beef samples. Still, the bacterial growth was suppressed to an extent deemed sufficient for extending the shelf life of the product. Since ethanol can be easily traced in meat products, it can be used as a marker of meat spoilage and testify whether the irradiation of meat was carried out correctly and homogeneously.

Summarizing all the results obtained during food irradiation research, it can be concluded that all VOCs are suitable markers of biochemical processes occurring in meat during storage: oxidative processes after irradiation, oxidation due to ROS_0_, which are always present in the product during storage, and microbial–enzymatic processes, to name a few. In this paragraph, we have highlighted only those VOCs that clearly showed a dose impact on the kinetics of VOC concentrations.

**Figure 9:**
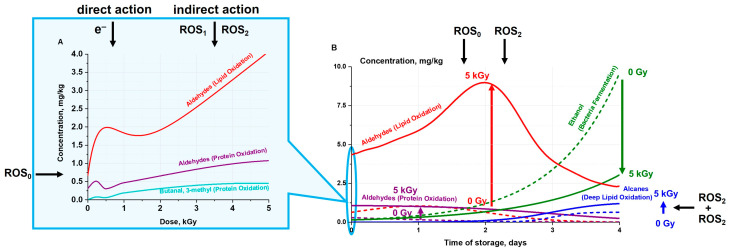
Dose dependencies of the concentrations of aldehydes, which are lipid oxidation products, as well as aldehydes resulting from the decomposition of proteins and aldehyde butanal,3-methyl recorded in the beef samples immediately after irradiation (**A**) and during four-day storage for the non-irradiated samples and for the samples irradiated with 5 kGy (**B**); dependencies of ethanol concentration and alkanes concentrations registered in the non-irradiated samples and in the samples irradiated with 5 kGy during four-day storage (**B**).

Figure 9 shows how irradiation markers appeared and disappeared throughout the four-day experiment. Figure 9A shows the dose dependencies of the concentrations of aldehydes formed from lipids (red curve), the concentrations of aldehydes formed from proteins (purple curve), and butanal,3-methyl aldehyde concentration (blue curve) measured immediately after irradiation. The dependencies shown in Figure 9A are calculated using Formula (5) and approximate the experimental data for aldehydes formed from lipids (Figure 6B) and aldehydes formed from proteins (Figure 6C). Figure 9A zooms into day 0 measurements of aldehyde concentrations shown in Figure 9B. As can be seen from Figure 8, dose dependencies of the concentrations of aldehydes, which are lipid oxidation derivatives, as well as aldehydes resulting from the decomposition of leucine and isoleucine amino acids, were recorded immediately after irradiation, indicating the impact of direct and indirect action of accelerated electrons through the effect of ROS_1_ on lipids and proteins (Figure 9A). With an increase in the irradiation dose, the rate of increase in lipid aldehyde concentration significantly exceeds the rate of increase in protein aldehyde concentrations, which a sign of a high sensitivity of lipids to irradiation within the dose range of 0.25 kGy to 5 kGy compared to the sensitivity of proteins.

As the storage time passed, secondary and tertiary ROS led to the development of lipid oxidation, which was detected by the peak in the concentration of aldehydes formed from lipids from day 1 to day 2, after which the concentration of aldehydes gradually subsided to the values of non-irradiated samples. On day 3 and day 4 of monitoring, lipid oxidation products formed multistage oxidative products, hydrocarbons, whose peak concentration was observed on day 3 and 4 of monitoring. At the same time, the development of microbial–enzymatic processes on ethanol concentration was recorded, which indicated an increase in the number of microorganisms in beef. Overall, as storage time passed, oxidation products occurring as a result of irradiation replaced each other, with the maximum dose–effect impact being observed on day 1 and day 2 after irradiation, while bacterial content growth intensified on day 3 and day 4 of storage of beef samples at 4 °C (Figure 9B). The aldehydes pentanal, hexanal, heptanal, octanal, and nonanal, and butanal,3-methyl, hydrocarbons, and ethanol found in irradiated beef during this study can serve as indicators of the impact of irradiation on beef: the content of VOCs can reveal the dose absorbed by the beef samples, as well as the ROS and microorganism concentration in beef after irradiation. Thus, the prolonged dose–effect impact of accelerated electrons on chemical parameters of meat during storage is crucial for selecting the optimal meat irradiation method.

### 2.5. Kinetics of the Concentration of Aldehydes in the Beef Samples Irradiated with Different Doses

We used concentrations of aldehydes pentanal, hexanal, heptanal, octanal, and nonanal, formed as a result of lipid oxidation to construct a mathematical model of the change in the aldehyde concentrations over time in the beef samples irradiated with different doses for developing a criterion for selection of optimal parameters of meat irradiation. Let us assume that the change in aldehyde concentration occurs as a result of both direct ionization of lipids by accelerated electrons and oxidation by ROS_1_ occurring as a result of water radiolysis in the beef samples. During the chemical stage of irradiation, interaction of lipids with ROS_1_ results in the formation of lipid peroxidation products hydroperoxides and lipid radicals, which continue to oxidize lipids throughout the observation time.

Let us assume that lipids are the main source of aldehydes, namely, lipid oxidation causes aldehyde to appear in the beef samples during storage, and aldehydes are initially absent in fresh tenderloin beef. Since background ROS_0_ can always be found in the stored beef samples as a result of the presence of oxygen, the aldehyde concentration in the non-irradiated samples was slightly above zero at the time of the first measurement. Let *l*(*t*) be the amount of non-oxidized lipids in the beef samples at time *t*; *α*, 1/s, be the rate constant of lipid oxidation due to both the direct action of radiation and the indirect action of ROS_1_ whose amount depends on the irradiation dose; let *R*_0_ be the number of ROS_0_, present in all beef samples during storage; let *β* be the coefficient of proportionality, namely, the number of ROS_1_ per unit of the dose absorbed by a beef sample.

Since the radical ${OH}^{*}$ is the most effective oxidant for lipids [47], it can be assumed that only a part of the formed ROS_1_ species is involved in lipid oxidation. Let *m* = *m*(*D*) be the fraction of ROS_1_ which oxidize lipids. Then the change in the number of non-oxidized lipids over time can be described by the following first order linear differential equation:

(6) $$\frac{dl}{dt}=-\alpha m\left(R_{0}+\beta D\right)l.$$

According to Equation (6), the amount of non-oxidized lipids decreases with time according to the exponential law:

(7) $$l\left(t\right)=l_{o}\left(D\right)e^{-\alpha m\left(R_{0}+\beta D\right)t},$$

where *l_o_* is the initial amount of non-oxidized lipids in meat samples before irradiation with accelerated electrons.

Based on the fact that during irradiation of meat, not all lipids *l_o_* were subjected to direct or indirect action of accelerated electrons, and also during storage not all lipids were oxidized under the action of organic radicals, the change in the number of oxidized lipids with time can be represented as:

(8) $$l^{ox}\left(t\right)=l_{o}k\left(1-e^{-\alpha m\left(R_{0}+\beta D\right)t}\right),$$

where *k* is the fraction of lipids that were affected by the direct ionization caused by accelerated electrons and also interacted with ROS_1_ formed in meat samples. Hence, *k = k*(*D*) depends on the irradiation dose, because the higher the dose, the longer the irradiation time and the more electrons interact with the lipids, as well as more reactive oxygen species being formed in the meat samples both during irradiation.

Let us denote the quantity of aldehydes in the beef samples as *x*. Since not all oxidized lipids form aldehydes, let us assume that only a fraction *q* of the total amount of oxidized lipids $l^{ox}$ converted to aldehydes. Since the rate of increase in aldehydes is proportional to the rate of the increase in the quantity of oxidized lipids, the rate of the increase in the amount of aldehydes can be expressed as follows:

(9) $$\frac{dx}{dt}=q\frac{dl^{ox}}{dt}.$$

Taking into account (9), the equation describing the increase in aldehyde concentration due to lipid oxidation can be represented as follows:

(10) $$\frac{dx}{dt}={ql}_{o}k\alpha m\left(R_{0}+\beta D\right)e^{-\alpha m\left(R_{0}+\beta D\right)t}.$$

According to the experimental data presented in Figure 6B, the concentration of aldehydes after peaking on day 1 and day 2 of monitoring declined during the last two days of observation. Thus, Equation (10) is transformed into the following inhomogeneous differential equation of the first order:

(11) $$\frac{dx}{dt}={ql}_{o}k\alpha m\left(R_{0}+\beta D\right)e^{-\alpha m\left(R_{0}+\beta D\right)t}-n\gamma x,$$

where *γ*, 1/s is the rate constant of aldehyde decomposition over time and *n* = *n*(*D*) is the fraction of aldehydes that decompose into other compounds. If solved by the standard method and taking into account that initially there are no aldehydes, Equation (11) can be represented as:

(12) $$x\left(t\right)=\frac{ql_{o}k\alpha m\left(R_{0}+\beta D\right)}{\alpha m-\gamma n}{(e}^{-\gamma nt}-e^{-\alpha m\left(R_{0}+\beta D\right)t}).$$

**Figure 10:**
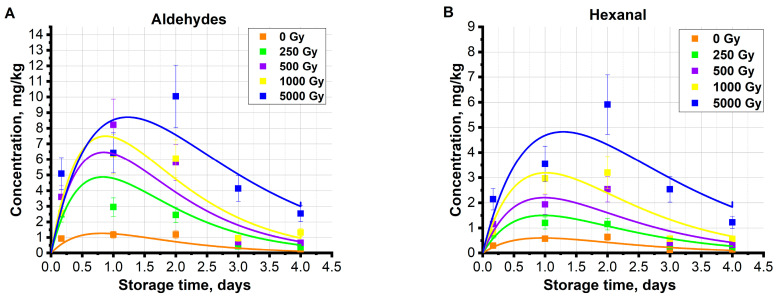
Storage time dependence of summary concentration of aldehydes *C_ald_* (**A**) and the concentration of hexanal *C_H_* (**B**) in the non-irradiated samples as well as in the samples irradiated with doses ranging from 0.25 kGy to 5 kGy, and approximation of experimental dependence using Formula (6) (**A**).

Figure 10A shows the experimental data of summary concentration of the aldehydes pentanal, hexanal, heptanal, octanal, and nonanal in the beef samples irradiated with doses ranging from 0.25 kGy to 5 kGy during the storage time. Figure 10A also presents an approximation of the experimental data calculated using Formula (12). In Figure 10B, the calculated relationship (12) is presented for the concentration of aldehyde hexanal *C_H_*, whose concentration mostly contributed to the total concentration of aldehydes produced by lipid oxidation.

According to the developed model, it was obtained that the expressions ${ql}_{0}k\left(D\right),\;\alpha m\left(D\right)\left(R_{0}+\beta D\right),\;\gamma n\left(D\right)$ are monotonic and dependent on the dose as follows:

(13) $${ql}_{0}k\left(D\right)=x_{1}+x_{2}\;*\left(1-e^{-x_{3}D}\right),$$

(14) $$\alpha m\left(D\right)\left(R_{0}+\beta D\right)=x_{4}+x_{5}D,$$

(15) $$n\left(D\right)=x_{6}+x_{7}D,$$

where $x_{1},x_{2}{,x}_{3,}x_{4},x_{5},x_{6},x_{7}$ are approximation coefficients with the values presented in Table 3. In the case of the total aldehyde concentration approximation, the values of the model parameters are averages of all aldehydes from lipids. The values of approximation parameters calculated for the total aldehyde concentration using Formula (12) are averaged throughout all aldehyde group.

High values of correlation coefficients of *R* = 0.98–0.99 indicate the adequacy of the proposed mathematical model of the temporal relationship of the concentration of aldehydes formed from lipids in the beef samples irradiated with different doses. According to the model, the quantity of oxidized lipids ${ql}_{0}k\left(D\right)$ that form aldehydes increases exponentially with an increase in the irradiation dose, which can be explained by the fact that the higher the dose, the more electrons directly acting on lipids as well as ROS_1_ occur as a result of water radiolysis, and the more lipids are oxidized during irradiation. According to the model, the amount of ROS, including background ROS_0_ and ROS_1_ appearing as a result of irradiation, expressed as *m*(*D*)$\left(R_{0}+\beta D\right)$, monotonically decreases with a higher irradiation dose, since a higher dose can increase the probability of the interaction of radicals with other organic molecules. Moreover, the chances of radical neutralization may be higher with the increase in the irradiation dose. As the model shows, the quantity of aldehydes that decompose into other compounds expressed as $\gamma n\left(D\right)$ decreases with a higher irradiation dose, which can be explained by the fact that the probability of aldehydes entering various chemical reactions that cause them to decompose is influenced by other biochemical processes occurring in irradiated beef during storage.

## 3. Materials and Methods

### 3.1. Methodology

Fresh ground tenderloin beef purchased at a local market was divided into 100 portions weighing 0.5 g each and placed into ∅ 9 mm 3.9 cm long Eppendorf-type microcentrifuge polypropylene tubes. Of the 100 beef samples, 20 were used as controls and 80 were irradiated with 0.5 μA beam current using a 1 MeV electron accelerator UELR-1-25-T-001 (SINP MSU, Moscow, Russia). Ground beef was laid out in a thin layer along the entire length of the tube such that its thickness did not exceed 2 mm to increase the uniformity of beef irradiation.

The samples were divided into 4 sets, 20 tubes in each, and each set was irradiated on the duralumin plate individually, in 2 sessions, 10 tubes in each, with 10 Gy/s at the ambient temperature of 20 °C, to ensure that each set received the doses of 250, 500, 1000 or 5000 Gy (Figure 1). To control the dose absorbed by beef samples, the time of exposure and the charge absorbed by the duralumin plate were recorded during each irradiation session. Immediately after irradiation, four tubes from each set including the non-irradiated set were subjected to gas chromatography–mass spectrometry (GC/MS) analysis to detect the changes in VOCs occurring in beef samples as a result of irradiation. The remaining 16 tubes from each set, placed in glass vials and stored at a temperature of 4 °C, were subjected to mass spectrometry every day during 4 subsequent days after irradiation. During 4 days after irradiation, both the irradiated and control samples were stored in the fridge at a temperature of 4 °C. Control non-irradiated samples were kept in the same conditions as the samples irradiated at different doses of electron irradiation.

We established that a four-day storage of beef samples at 4 °C maximizes the effectiveness of volatile compound observations, because clear dose dependencies manifest themselves while bacterial activity in chilled meat is relatively mild. Storage of meat for five days and longer has a detrimental effect on the composition of organic compounds as a result of an intensive bacterial activity. Since four-day monitoring allows the establishment of clear dose dependencies of volatile compound concentrations, it is feasible to look for reliable dose-dependent markers during this time frame.

### 3.2. Dosimetry

A Geant 4 toolkit (CERN, European Organization for Nuclear Research, Geneva, Switzerland) was used in the study to calculate the dose absorbed by the beef samples during irradiation. To simulate the electron irradiation of ground beef, the samples, represented by 39 mm × 9 mm × 2 mm water parallelepipeds, were irradiated with 10^10^ electrons taking into account the irradiation method applied to the beef samples and the energy spectrum of the 1 MeV electron accelerator UELR-1-25-T-001 [9]. The source of electrons was represented by a 100 mm × 100 mm square placed 12 cm far from the duralumin plate holding water parallelepipeds.

The calculation of the dose absorbed by the samples was carried out taking into account the charge absorbed by the samples, beam current and irradiation time according to the algorithm described in [9].

### 3.3. GC/MS Analysis

Ground beef samples were subjected to GC/MS analysis without prior extraction to ensure that the chemical yield of volatile organic compounds was determined only by biochemical composition of the beef samples without being impacted by the chemical composition of a solution. Immediately after irradiation, ground beef from each of two polypropylene tubes irradiated at the same dose was placed in 20 mL glass vials (Shimadzu, Kyoto, Japan), and tightly sealed with a lid. A total of 50 vials were prepared, 10 vials for each irradiation dose, including 10 vials with non-irradiated samples. The sealed vials that were not analyzed immediately after irradiation were stored throughout the study for 4 days at 4 °C (Figure 1).

During chemical analysis, beef samples were thermostated for 20 min at 90 °C, then 1 mL of the vapor phase was injected into a Shimadzu GC/MS-QP2010 Ultra (Shimadzu, Japan) chromatograph equipped with an HT200H Headspace Autosampler (NTA, Avola, Italy) to detect volatile organic compounds in the ground beef samples. Measurements for each irradiation dose were performed twice on each day of monitoring.

During the GC/MS analysis, VOCs were separated using a 60 m × 0.32 mm × 1.8 μm VF-624 capillary column (Agilent, Santa Clara, CA, USA). The temperature regime for separation of the compounds was automatically selected to ensure an efficient separation of volatile compounds with a similar molecular weight. The initial temperature at which the compounds were processed was 40 °C. Then, the samples were kept for 5 min at 40 °C, and then the temperature was raised to 220 °C at a rate of 6 °C/min during 30 min, and then the detection of VOCs emitted by the beef samples was carried out at a temperature of 220 °C for 5 min. Helium was used as carrier gas at a flow rate of 1.5 mL/min through the capillary column. The mass spectrometric detector used in the GC/MS analysis had an electron impact mode of 70 eV with a quadrupole temperature of 200 °C, and ion source temperature of 230 °C. The chromatograms were recorded in the scan mode for all ions, with *m*/*z* values ranging from 33 to 350 at a speed of 3.3 scans per second.

### 3.4. Statistical Analysis

The VOCs were identified by comparing their mass spectra with the spectra from the NIST/EPA/NIH Mass Spectral Library 2008 (NIST 08) using GC/MS solution version 2.70 software (Shimazu, Japan).

The concentrations of volatile compounds (mg/kg) were calculated taking into account the calibration relationships obtained using the chromatographic analysis of standard volatile compound samples, peak area on the chromatogram and the original sample weight.

The standard addition method was used to enhance the precision of data on the VOC content in the beef samples. The calibration dependence for the determination of the VOC concentrations was built as follows: 1 µL to 100 µL aliquots of solutions of c were added to a 20 mL vial with the control beef sample weighing 1.00 g. The vials were tightly sealed and stored for one hour at room temperature before performing GC/MS analysis according to the conditions described in Section 3.3. Metrological characteristics were evaluated according to ISO 17025 [48]. Table 4 shows CAS numbers and the purity of the aliquots of solutions of standard VOC samples.

The content of standard VOC samples in the spiked samples was selected in such a way as to cover the whole range of VOC content in the studied beef samples. For example, since the concentration of acetaldehyde ranged from 19 mg/kg to 25 mg/kg in all the beef samples, the study assumed 30 mg/kg as the upper limit to ensure that the entire range was fully covered. For other VOCs ranging within 2–10 mg/kg, the upper limit was 10 mg/kg. Assuming that the signal-to-noise ratio values for each VOC standard sample was 3 for MDL and 10 for MQL, the MQL values ranged from 0.005 mg/kg to 0.056 mg/kg and MDL values ranged from 0.02–0.2 mg/kg for all VOCs. The RSD values for n = 6 replicate measurements for all VOCs were obtained by the same instrument operator within one day and ranged from 5.8% to 18%. To verify the accuracy of VOCs found in the beef samples during the study, three different concentrations of standard VOC samples were used as a reference, and the measurements were performed three times for all concentrations to enhance the precision of calculations. As a result, the accuracy of VOC determination for a vast majority of compounds was above 90%, with the exception of acetaldehyde, dimethyl sulfide, acetone and propanal,2-methyl-, which have a low boiling point, whose accuracy ranged from 77% to 85% due to the loss of VOCs during the separation.

**Figure 11:**
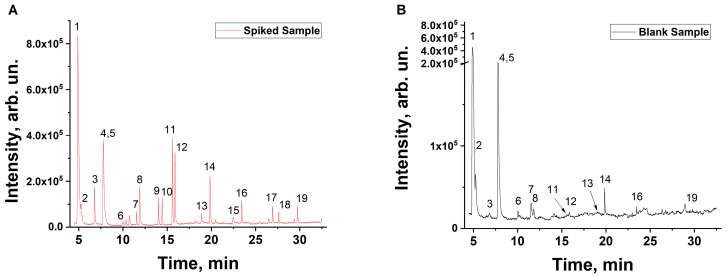
Chromatograms of non-irradiated control sample (**A**) and the sample with the addition of standard VOC samples (**B**). The concentration of acetaldehyde is 20 mg/kg, the concentration of other standard samples is 1 mg/kg. Compounds identified in the beef samples: 1—acetaldehyde, 2—methanethiol, 3—ethanol, 4—acetone, 5—dimethyl sulfide, 6—propanal,2-methyl-, 7—2,3-butandione, 8—2-butanone, 9—butanal,3-methyl-, 10—butanal,2-methyl-, 11—2-pentanone, 12—pentanal, 13—1-pentanol, 14—hexanal, 15—1-hexanol, 16—heptanal, 17—octanal, 18—1-hexanol,2-ethyl-, 19—nonanal.

Chromatograms of non-irradiated control beef sample and the sample with the addition of standard VOC samples are shown in Figure 11.

The VOC validation parameters are represented in Table 5.

Statistical and analytical processing of the VOC concentrations was performed using Microsoft Excel v16.0 (Microsoft, Redmond, WA, USA) and OriginPro 2018 SR1 b.9.5.1.195 (Origin Lab Corporation, Northampton, MA, USA). To construct a heat map of the VOC profile, as well as approximating curves describing the behavior of the compounds in the samples irradiated with different doses over time, MATLAB R2023b v23.2.0.2365128 (Mathworks, Natick, MA, USA) was used.

## 4. Conclusions

During a four-day monitoring of the chilled beef samples irradiated with accelerated electrons at a dose ranging from 0.25 kGy to 5 kGy, it was found that the chemical yield of volatile organic compounds (VOCs) depended nonlinearly on the irradiation dose due to complex mutual transformation of VOCs of different classes. The analysis of the dose-dependent relationship of VOC concentrations revealed that lipid oxidation-derived aldehydes and protein oxidation-derived aldehydes showed different chemical yield and accumulation rate immediately after irradiation. While intensive lipid oxidation was triggered by the dose of 250 Gy and intensified with an increase in the irradiation dose, the protein oxidation rate became commensurable with the lipid oxidation rate for the beef samples irradiated with 5 kGy.

Since the dose impact on mutual transformation of VOCs in the chilled beef samples persisted for some time after irradiation, it is necessary to consider not only the initial readings of VOCs upon irradiation but also to take into account the behavior of VOCs in the stored samples over time. Lipid oxidation and protein oxidation markers as well as markers of bacterial activity could help to establish the criteria for determining the effective dose range: while the lowest limit of the dose range can be determined by the concentration of ethanol, the highest limit of the dose range can be established by the concentrations of lipid oxidation-derived aldehydes and protein oxidation-derived aldehydes. To conclude, under the conditions of our experiment, the doses ranging from 0.25 kGy to 1 kGy have proved to be most effective for beef irradiation with accelerated electrons, since this dose range decreases the bacterial content without considerable irreversible changes in chemical composition of chilled beef during storage.

During the experiment, we had to take into consideration a few limitations, which inevitably occur due a complex and variable composition of meat as well as the complexity of biochemical processes, such as bacterial and enzymatic activity, oxidation due to reaction oxygen species, and other processes acting upon each other during storage. Considering that compounds with *m*/*z* values below and above the range studied in this paper also occur in irradiated biological objects, our future study will use a highly sensitive solid-phase microextraction technique to expand the range of volatile compounds that can be reliably detected in biological samples after irradiation. We are also planning to use liquid chromatography–mass spectrometry/mass spectrometry to find dose-dependent markers among non-volatile organic compounds in irradiated food samples.

## Figures and Tables

**Table 1:** Concentration of the identified compounds in the beef samples analyzed one hour after irradiation (mg/kg); n = 3—number of repeats, relative error was 20% with confidence level *p* = 0.95.

Compound	Retention Time (min)	Dose (kGy)					Trend *
		0	0.25	0.5	1	5	
Acetaldehyde (1) ***	4.88	24.8	23.45	20.9	19.4	25.0	↓↑
Methanethiol (2)	5.23	2.95	2.3	2.3	2.0	2.0	↓
Ethanol (3)	6.80	0.033	0.11	0.061	0.034	0.18	↑
Acetone (4)	7.77	0.74	0.74	0.58	0.67	0.77	—
Dimethyl sulfide (5)	7.93	0.12	0.084	0.090	0.11	0.22	↓↑
Propanal,2-methyl- (6)	10.02	0.31	0.43	0.24	0.28	0.62	↑
2,3-butandione (7)	11.51	0.50	0.99	0.74	0.72	0.93	↑
2-butanone (8)	11.80	0.17	0.29	0.25	0.44	1.7	↑
Butanal,3-methyl- (9)	14.12	ND **	0.08	0.062	0.19	0.45	↑
Butanal,2-methyl- (10)	14.43	ND	0.06	ND	ND	0.30	↑↓↑
2-pentanone (11)	15.49	0.004	0.011	0.005	0.006	0.015	↑
Pentanal (12)	15.84	0.031	0.084	0.17	0.12	0.19	↑
1-pentanol (13)	18.90	0.081	0.14	0.18	0.17	0.37	↑
Hexanal (14)	19.83	0.30	0.75	1.2	0.83	2.2	↑
1-hexanol (15)	22.48	ND	0.086	0.11	0.069	0.16	↑
Heptanal (16)	23.45	0.20	0.67	1.3	0.81	1.1	↑
Octanal (17)	26.75	ND	0.27	0.38	0.24	0.43	↑
1-hexanol,2-ethyl- (18)	27.54	ND	0.13	0.16	ND	ND	↑↓
Nonanal (19)	29.80	0.095	0.28	0.39	0.30	0.56	↑

* Trend ↑ means increase, ↓ means decrease, ↓↑ means decrease then increase, — no change with an increase in the dose. ** ND—not detected. *** The numbers in the parentheses next to each compound indicate peak positions on the chromatogram (Figure 3).

**Table 2:** Function parameters for different VOCs.

Compound	*k_A_* (kGy^−1^)	*k_B_* (kGy^−1^)	$C_{0}^{A}$ (mg/kg)	$C_{0}^{B}$ (mg/kg)	*g* (kGy^−1^)	*R*	SE
Hexanal	2.22	2.22	1.89	0.27	0.43	0.99	0.22
1-pentanol	1.77	1.77	0.28	0.08	0.07	1.00	0.01
Dimethyl sulfide	0.01	37.1	362.1	0.12	0.03	1.00	0.01
2-butanone	1.34	335.2	64.6	0.17	0.34	1.00	0.06

**Table 3:** Approximation coefficients calculated using Formulas (13)–(15).

Compound	$x_{1}$ (rel.un.)	$x_{2}$ (rel.un.)	$x_{3}{(\mathrm{kGy}}^{-1})$	$x_{4}$ (rel.un.)	$x_{5}({\mathrm{kGy}}^{-1})$	$x_{6}$ (rel.un.)	$x_{7}({\mathrm{kGy}}^{-1})$	*R*
Hexanal	1.67	11.41	0.98	1.02	−0.048	1.05	−0.0571	0.98
All Aldehydes	3.66	18.04	3.37	1.16	−0.054	1.29	−0.1097	0.99

**Table 4:** CAS numbers and the purity of the aliquots of solutions of standard VOC samples.

Compound	CAS Number	Purity	Manufacturer
Acetaldehyde	75-07-0	99%	PanReac, Barcelona, Spain
Ethanol	64-17-5	Analytical standard	Sigma Aldrich, St. Louis, MO, USA
Acetone	67-64-1	Analytical standard	Sigma Aldrich, USA
Dimethyl sulfide	75-18-3	Analytical standard	Sigma Aldrich, USA
Propanal,2-methyl-	78-84-2	>98.0% (GC)	TCI, Tokyo, Japan
2,3-butandione	431-03-8	99.0%	Acros Organics, Geel, Belgium
2-butanone	78-93-3	For spectrophotometry	TCI, Japan
Butanal,3-methyl-	290-86-3	Analytical standard	Sigma Aldrich, USA
Butanal,2-methyl-	96-17-3	≥95%, FG	Sigma Aldrich, USA
2-pentanone	107-87-9	Analytical standard	Sigma Aldrich, USA
Pentanal	110-62-3	Analytical standard	Sigma Aldrich, USA
1-pentanol	71-41-0	99%, pure	Acros Organics, Belgium
Hexanal	66-25-1	Analytical standard	Sigma Aldrich, USA
1-hexanol	111-27-3	standard for GC	Sigma Aldrich, USA
Heptanal	111-71-7	Analytical standard	Sigma Aldrich, USA
Octanal	124-13-0	For synthesis (99%)	Sigma Aldrich, USA
Hexanol,2-ethyl	104-76-7	≥99.6%	Sigma Aldrich, USA
Hexane	110-54-3	Analytical standard	Sigma Aldrich, USA
Heptane	142-82-5	suitable for HPLC, ≥99%	Sigma Aldrich, USA
Octane	111-65-9	Analytical standard	Sigma Aldrich, USA
2-butanone, 3-hydroxy-	513-86-0	For synthesis (99%)	Sigma Aldrich, USA
Butanol,3-methyl-	123-51-3	ACS, 98.5%	Acros Organics, Belgium
Butanol,2-methyl-	3391-86-4	98%	Acros Organics, Belgium
Propanol,2-methyl-	78-83-1	99+%, For spectrophotometry	Acros Organics, Belgium
1-octen-3-ol	3391-86-4	98%	Acros Organics, Belgium
Nonanal	124-19-6	For synthesis (99%)	Sigma Aldrich, USA

**Table 5:** VOC validation parameters.

Compound	Linearity Range (mg/kg)	RSD % (n = 6)	MDL (mg/kg)	MQL (mg/kg)	Accuracy %
Acetaldehyde	0.2–30	16	0.056	0.2	80
Ethanol	0.03–10	13	0.009	0.03	92
Acetone	0.2–10	15	0.050	0.2	85
Dimethyl sulfide	0.08–10	18	0.021	0.08	77
Propanal,2-methyl	0.05–10	14	0.017	0.05	82
2,3-butandione	0.1–5	8.5	0.025	0.1	93
2-butanone	0.05–10	7.6	0.015	0.05	94
Butanal,3-methyl	0.05–5	13	0.013	0.05	92
Butanal,2-methyl	0.05–5	12	0.014	0.05	92
2-pentanone	0.05–10	7.2	0.014	0.05	94
Pentanal	0.03–10	8.1	0.010	0.03	95
1-pentanol	0.08–10	5.8	0.022	0.08	93
Hexanal	0.03–10	7.8	0.010	0.03	96
1-hexanol	0.06–10	6.5	0.017	0.06	93
Heptanal	0.05–10	7.4	0.013	0.05	95
Octanal	0.1–5	6.2	0.026	0.1	92
Hexanol, 2-ethyl	0.1–10	8.2	0.025	0.1	105
Hexane	0.02–2	12	0.0052	0.02	89
Heptane	0.02–2	12	0.0053	0.02	90
Octane	0.02–2	11	0.0050	0.02	96
2-butanone,3-hydroxy-	0.1–5	9.2	0.026	0.1	105
Butanol,3-methyl	0.08–10	7.5	0.023	0.08	104
Butanol,2-methyl	0.08–10	7.7	0.023	0.08	103
Propanol,2-methyl	0.1–10	8.1	0.025	0.1	91
1-octen-3-ol	0.1–4.5	12	0.025	0.1	104
Nonanal	0.06–10	9.7	0.017	0.06	96

## Data Availability

Data are contained within the article and Supplementary Materials.

## Supplementary Materials

The following supporting information can be downloaded at https://www.mdpi.com/article/10.3390/molecules29050940/s1. Table S1: Concentration of the identified compounds in the beef samples analyzed after irradiation during 4 days of storage at 4 °C (mg/kg); n = 3—number of repeats, relative error was 20% with confidence level *p* = 0.95.
