# Supplementary material for: Volatile Compound Markers in Beef Irradiated with Accelerated Electrons
Source: Molecules. 2024 Feb 21;29(5):940. doi: 10.3390/molecules29050940 (PMC10933855; doi:10.3390/molecules29050940)
Supplement: Supplementary file 1 [file molecules-29-00940-s001.zip › molecules-2848406-supplementary.pdf]

Table S1. Concentration of the identified compounds in the beef samples analyzed after irradiation during 4 days of storage at 4 °C (mg/kg); n=3 - number of repeats, relative error was 20% with confidence level P=0.95.

Category	Compound	Dose (kGy)	Storage time (day)				
			0	1	2	3	4
Aldehydes	Acetaldehyde	0	24.8	8.8	2.9	045	0.67
		0.25	23.45	15.6	10.9	0.073	0.85
		0.5	20.9	21.1	14.7	0.41	0.95
		1	19.4	17.9	3.0	0.42	0.54
		5	25.0	15.4	18.7	4.1	1.1
	Propanal, 2-methyl-	0	0.31	0.14	ND	ND	ND
		0.25	0.43	0.31	ND	ND	ND
		0.5	0.24	0.38	0.18	ND	ND
		1	0.28	0.31	0.19	ND	ND
		5	0.62	0.52	0.48	0.18	0.2
	Butanal, 3-methyl-	0	ND	ND	ND	ND	ND
		0.25	0.08	ND	ND	ND	ND
		0.5	0.062	0.14	ND	ND	ND
		1	0.19	0.22	ND	ND	ND
		5	0.45	0.28	0.48	0.33	0.14
	Pentanal	0	0.031	0.053	0.037	ND	0.0042
		0.25	0.084	0.12	0.092	0.017	0.011
		0.5	0.17	0.19	0.27	ND	0.021
		1	0.12	0.31	0.29	0.02	0.038
		5	0.19	0.24	0.42	0.11	0.055
	Hexanal	0	0.30	0.57	0.64	0.066	0.14
		0.25	0.75	1.2	1.2	0.27	0.22
		0.5	1.2	1.9	2.5	0.41	0.33
		1	0.83	3.0	3.2	0.57	0.57
		5	2.2	3.5	6.9	2.5	1.2
	Heptanal	0	0.20	0.33	0.23	0.45	0.097
		0.25	0.67	0.8	0.66	0.14	0.15
		0.5	1.3	1.2	1.8	0.19	0.22
		1	0.81	1.7	2.1	0.22	0.39
		5	1.1	1.2	1.9	0.72	0.5
	Octanal	0	ND	0.045	0.14	ND	ND
		0.25	0.27	0.31	0.3	ND	ND
		0.5	0.38	3.7	0.59	ND	ND
		1	0.24	0.58	0.67	ND	0.11
		5	0.43	0.43	0.72	0.26	0.24
	Nonanal	0	0.095	0.033	0.15	ND	ND
		0.25	0.28	0.21	0.23	ND	ND
		0.5	0.39	0.77	0.48	0.10	0.091
		1	0.3	0.44	0.58	0.15	0.21
		5	0.56	0.47	0.71	0.34	0.31

Alcohols	Ethanol	0	0.033	0.044	0.54	2.3	9.4
		0.25	0.11	0.088	0.31	1.4	4.6
		0.5	0.061	0.056	0.17	2.1	3.2
		1	0.034	0.13	0.18	1.9	6.4
		5	0.18	0.17	0.12	1.1	3.0
	1-butanol, 3-methyl-	0	ND	ND	1.4	0.6	1.5
		0.25	ND	ND	1.2	1.7	1.1
		0.5	ND	ND	0.82	1.2	0.69
		1	ND	ND	1.2	1.5	1.3
		5	ND	ND	ND	2.0	1.5
	1-butanol, 2-methyl-	0	ND	ND	0.2	ND	0.25
		0.25	ND	ND	0.25	ND	0.19
		0.5	ND	ND	0.38	0.18	0.13
		1	ND	ND	0.77	0.33	0.18
		5	ND	0.03	0.27	0.5	0.33
	1-propanol, 2-methyl-	0	ND	ND	0.26	0.14	0.38
		0.25	ND	ND	0.19	0.21	0.16
		0.5	ND	ND	ND	0.22	0.14
		1	ND	ND	0.086	0.38	0.40
		5	ND	ND	ND	0.55	0.60
	1-pentanol	0	0.081	0.14	0.47	0.085	0.25
		0.25	0.14	0.28	0.56	0.26	0.26
		0.5	0.18	0.29	0.68	0.41	0.18
		1	0.17	0.64	0.90	0.43	0.50
		5	0.37	0.42	1.0	0.81	0.82
	1-hexanol	0	ND	0.034	0.59	0.1	0.33
		0.25	0.086	0.094	0.34	0.42	0.50
		0.5	0.11	0.087	ND	0.48	0.37
		1	0.069	0.20	0.51	0.54	0.94
		5	0.16	0.13	0.44	1.6	2.2
	1-octen-3-ol	0	ND	ND	0.25	0.14	ND
		0.25	ND	ND	0.16	0.18	0.43
		0.5	ND	ND	0.23	0.32	0.15
		1	ND	ND	0.79	0.3	0.35
		5	ND	ND	1.5	1.3	0.66
	1-hexanol, 2-ethyl-	0	ND	ND	0.36	0.098	0.36
		0.25	0.13	0.088	0.12	0.28	0.46
		0.5	0.16	ND	ND	0.38	0.093
		1	ND	0.14	0.18	0.16	0.10
		5	ND	0.096	0.27	0.24	0.18
Ketones	Acetone	0	0.74	0.4	0.42	0.023	0.07
		0.25	0.74	0.6	0.60	0.059	0.036
		0.5	0.58	0.76	0.63	0.095	0.035
		1	0.67	0.68	0.63	0.13	0.066
		5	0.77	0.56	0.86	0.47	0.36
	2,3-butandione	0	0.50	1.1	15.8	0.48	1.1
		0.25	0.99	0.80	22.51	1.1	0.59
		0.5	0.74	0.98	15.9	2.5	0.53
		1	0.72	1.1	11.2	2.7	1.1

		5	0.93	0.83	2.5	11.3	3.5
		0	0.17	0.083	ND	ND	ND
		0.25	0.29	0.19	ND	ND	ND
	2-butanone	0.5	0.25	0.37	ND	ND	ND
		1	0.44	0.39	0.37	ND	ND
		5	1.7	1.1	1.7	ND	ND
		0	0.004	ND	0.031	0.014	0.026
		0.25	0.011	ND	ND	0.018	0.02
	2-pentanone	0.5	0.005	ND	0.018	0.026	0.018
		1	0.006	ND	ND	ND	0.03
		5	0.015	ND	0.02	0.054	0.057
		0	ND	ND	18.6	0.26	0.21
		0.25	ND	ND	15.1	3.5	0.63
	2-butanone, 3-hydroxy--	0.5	ND	ND	11.6	0.95	0.19
		1	ND	ND	13.4	2.8	ND
		5	ND	ND	0.81	17.0	1.0
		0	ND	ND	2.9	0.30	0.52
		0.25	ND	ND	0.49	0.51	0.28
	2-butanone, 4-hydroxy-	0.5	ND	ND	0.50	0.91	0.46
		1	ND	ND	ND	3.5	1.7
		5	ND	ND	ND	9.7	7.9
Sulphurous Compounds		0	2.95	1.1	0.27	0.19	0.11
		0.25	2.3	2.1	0.47	0.95	1.4
	Methanethiol	0.5	2.3	3.1	1.4	1.0	0.61
		1	2.0	2.1	1.2	0.70	0.20
		5	2.0	1.9	3.2	0.44	0.66
		0	0.12	0.061	0.13	0.053	0.13
		0.25	0.084	0.087	0.09	0.055	0.083
	Dimethyl sulfide	0.5	0.09	0.089	0.12	0.081	0.074
		1	0.11	0.12	0.12	0.086	0.12
		5	0.22	0.12	0.14	0.15	0.19
Hydrocarbons (Alkanes)		0	ND	ND	ND	ND	0.029
		0.25	ND	ND	ND	ND	0.023
	Hexane	0.5	ND	ND	ND	0.023	0.031
		1	ND	ND	ND	0.036	0.045
		5	ND	0.026	0.0088	0.026	0.054
		0	ND	ND	0.16	0.11	0.36
		0.25	ND	ND	0.12	0.32	0.34
	Heptane	0.5	ND	0.08	0.16	0.30	0.40
		1	ND	0.14	0.25	0.29	0.53
		5	ND	0.24	0.81	1.1	0.82
		0	ND	ND	ND	0.037	0.17
		0.25	ND	ND	ND	0.14	0.15
	Octane	0.5	ND	ND	0.046	0.21	0.29
		1	ND	ND	0.017	0.17	0.32
		5	ND	0.11	0.10	0.17	0.33

	0	30.3	12.9	46.4	5.9	16.1
	0.25	30.8	22.9	55.9	11.6	12.6
Total VOCs	0.5	29.0	35.2	53.2	12.4	9,2
	1	26.4	30.0	41.8	17.3	16.2
	5	36.9	27.8	44.2	57.1	28.0

ND—Not Detected
